# TRPV6 calcium channel directs homeostasis of the mammary epithelial sheets and controls epithelial mesenchymal transition

**DOI:** 10.1038/s41598-020-71645-z

**Published:** 2020-09-07

**Authors:** Tytti Kärki, Eeva Kaisa Rajakylä, Anna Acheva, Sari Tojkander

**Affiliations:** 1 Section of Pathology, Department of Veterinary Biosciences, University of Helsinki, Agnes Sjöberginkatu 2, 00014 Helsinki, Finland; 2 Department of Applied Physics, Aalto University School of Science, Puumiehenkuja 2, 02150 Espoo, Finland

**Keywords:** Cancer, Cell biology, Molecular biology

## Abstract

Epithelial integrity is lost upon cancer progression as cancer cells detach from the primary tumor site and start to invade to the surrounding tissues. Invasive cancers of epithelial origin often express altered levels of TRP-family cation channels. Upregulation of TRPV6 Ca^2+^-channel has been associated with a number of human malignancies and its high expression in breast cancer has been linked to both proliferation and invasive disease. The mechanisms behind the potential of TRPV6 to induce invasive progression have, however, not been well elucidated. Here we show that TRPV6 is connected to both E-cadherin-based adherens junctions and intracellular cytoskeletal structures. Loss of TRPV6 from normal mammary epithelial cells led to disruption of epithelial integrity and abnormal 3D-mammo sphere morphology. Furthermore, expression level of TRPV6 was tightly linked to the levels of common EMT markers, suggesting that TRPV6 may have a role in the mesenchymal invasion of breast cancer cells. Thus, either too low or too high TRPV6 levels compromise homeostasis of the mammary epithelial sheets and may promote the progression of pathophysiological conditions.

## Introduction

Epithelial tissues form dynamic sheet-like barriers to enclose functionally distinct units in living organisms. Integrity and homeostasis of the epithelial sheets are crucial for normal body functions and compromised in several pathophysiological conditions. To adopt to various external cues, cell–cell junctions, connecting the neighboring cells in sheets, are sensing these external cues and modified accordingly to adjust their strength and permeability^1,2^. From all the cell junction types, intercellular cadherin-based adhesions, i.e. adherens junctions (AJs) are the major sites for sensing biophysical changes of the environment and can respond to mechanical forces to subsequently reinforce cellular contacts and intracellular cytoskeletal structures^3^. Contractile actomyosin bundles, lining the AJs, provide tension to support the junctions and their dynamics is maintained by several parallel upstream signaling cascades, including Rho/ROCK pathway, that insure the integrity of the cell–cell junctions^3–5^. Calcium has been shown to play a major role in the regulation of different types of actin-based structures, and act upstream of several kinases, regulating actin dynamics^6^. Local Ca^2+^-influxes could thus play a major role in the spatio-temporal control of peripheral actomyosin bundles and several recent papers have also linked mechanosensitive Ca^2+^-ion channels to the regulation of actomyosin dynamics^7–12^. However, most of these studies have concentrated on single migrating cells and the role of specific Ca^2+^-channels in the maintenance of actomyosin structures in epithelial sheets has remained unexplored.

One major ion channel family on the plasma membrane is formed by Transient receptor potential (TRP) channel superfamily, which is composed of a diverse group of proteins with variable selectivity for specific cations^13,14^. These cation channels are built by six transmembrane proteins and participate in the induction of several intracellular signaling cascades upon various cues. As they act in the crossroads of cellular microenvironment and central intracellular regulatory pathways, it is obvious that these channel proteins also play a role in the physiopathology of various diseases, including cancer^14,15^.

TRPV6 belongs to the vanilloid subfamily of TRP superfamily and besides closely related TRPV5, is highly calcium-selective in comparison to other TRP-family channel proteins^16–18^. Additionally, both TRPV5 and TRPV6 are constitutively active and their major function seems to be in the regulation of the whole body Ca^2+^-homeostasis as they are widely expressed in different Ca^2+^-absorbing epithelial tissue types^16,18^. In line with this, TRPV6 KO mice exhibit various physiological problems, including defective intestinal Ca^2+^-absorption, excessive Ca^2+^-excretion through the urinary tract as well as fertility problems^16–20^. Despite the central role in calcium homeostasis, the downstream pathways of this channel protein have remained poorly explored.

Overexpression of TRPV6 has been linked to several cancers of epithelial origin, including prostate, breast, ovary, colon and pancreatic cancers^18,21–23^. The mechanisms behind this phenomenon are, however, poorly understood: TRPV6 gene amplification is responsible for only under 1% of the breast carcinoma cases with high TRPV6 expression. Besides that, hormonal factors in an organ-dependent manner, may contribute to TRPV6 levels in some cancer types, as *trpv6* gene promoter contains estrogen receptor (ER) responsive element^24^. More rarely, some carcinomas, including cervical squamous cell carcinoma, show downregulation of TRPV6^25^. In breast carcinomas, overexpression of TRPV6 is a common event and the levels of this protein have been shown to be highly elevated, especially in the invasive regions^22,26^. High TRPV6 also correlates with poor prognosis in estrogen receptor-negative breast cancers^27^. This might be linked to TRPV6′s ability to promote cell proliferation through Ca^2+^-dependent calmodulin/calcineurin/NFAT pathway^18,26,28–30^. Activation of this pathway impacts genes involved in cell proliferation, viability and matrix degradation through MMPs^30,31^. Additionally, high TRPV6 is linked to increased levels of anti-apoptotic Bcl-2^32^ and downregulation of TRPV6 with specific siRNAs in pancreatic cancer cells led to significant decrease in Bcl-2, concomitant with triggered apoptosis^33^. Based on these previous studies, TRPV6 thus clearly plays a role in cell proliferation and survival. However, the other intracellular pathways, downstream of TRPV6, are poorly studied and their significance for cancer progression is not understood.

In this study we propose a novel role for TRPV6 in the regulation of breast epithelial homeostasis. Our studies in mammary epithelial cell lines demonstrate that TRPV6 is associated with E-cadherin mechanotransduction complex at the cell–cell junctions and that TRPV6 is expressed and recruited to the cell surface in a confluency-dependent manner. Depletion of TRPV6 caused disruption of the junction-maintaining peripheral actomyosin bundles possibly due to deregulation of the myosin light chain phosphorylation. Additionally, we show that TRPV6 levels tightly correlate with the expression of epithelial mesenchymal transition (EMT)-linked factors and depletion of TRPV6 from mammary epithelial or breast carcinoma cell lines leads to downregulation of several EMT-associated proteins. In all, our data provide novel information on the role of TRPV6 in normal epithelial homeostasis and a possible mechanism for its ability to provoke invasive progression.

## Results

### TRPV6 is expressed and recruited to the cell–cell junctions in a tension-sensitive manner

Previous studies have demonstrated that TRPV6 plays an important role in the Ca^2+^-influx of epithelial cells^16,20,34^. However, its role in the homeostasis of various epithelial tissues has remained poorly studied.

**Figure 1:**
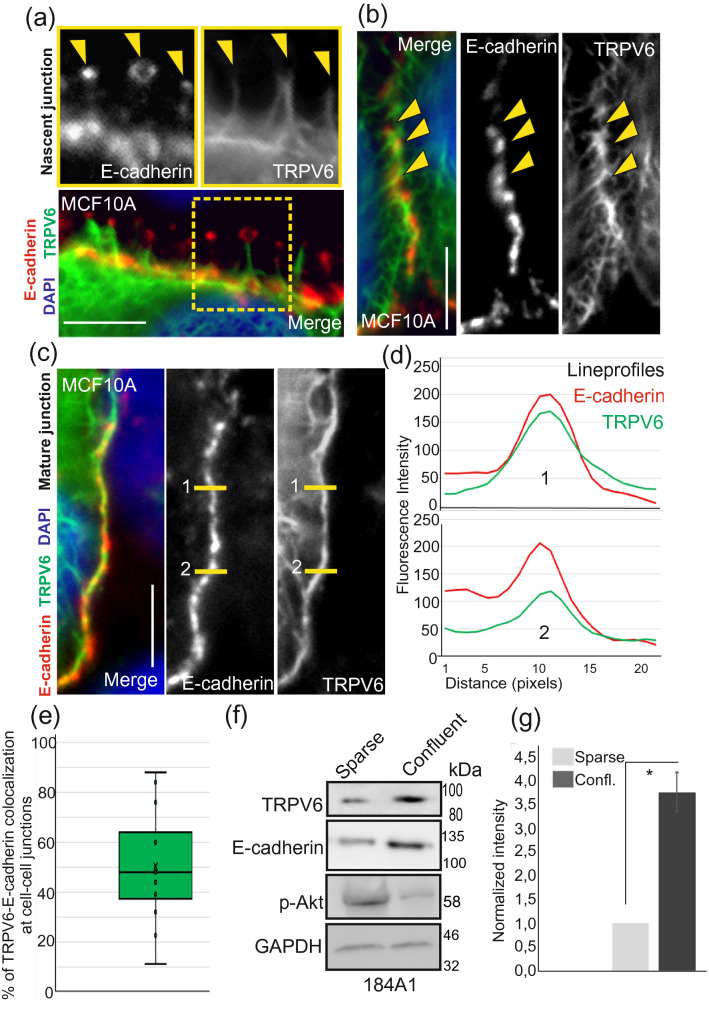
TRPV6 associates with E-cadherin-based cell–cell adhesions and is expressed in a confluency-dependent manner. (**a**) Semiconfluent MCF10A cells were fixed with methanol and specific antibodies against E-cadherin and TRPV6 were used in immunofluorescence stainings. A representative image from early cell–cell contacts shows that TRPV6 is linked to E-cadherin-based adhesions at the finger-like protrusions of nascent junctions (indicated with yellow arrows). Bar 10 μm. (**b**) Accumulation of TRPV6 at cell–cell contacts. Semi-confluent MCF10A cells were fixed with methanol and specific antibodies against TRPV6 and E-cadherin were used in immunofluorescence stainings. DAPI was used to visualize the nuclei. In semi-confluent cultures TRPV6 is linked but not colocalizing with E-cadherin. Bar 10 μm. (**c**) Accumulation of TRPV6 at cell–cell contacts. Confluent MCF10A cells were fixed with methanol and specific antibodies against TRPV6 and E-cadherin were used in immunofluorescence stainings. DAPI was used to visualize the nuclei. At more mature cell–cell junctions TRPV6 shows colocalization with E-cadherin. Bar 10 µm. (**d**) Lineprofiles, related to (**c**). Lineprofiles were analyzed in ImageJ and they show representative colocalization of TRPV6 and E-cadherin at the cell–cell contacts. (**e**) Percentage of E-cadherin and TRPV6 colocalization. E-cadherin staining at the cell–cell junctions was used as a ROI, region of interest. Percentage of TRPV6 at this ROI was measured in ImageJ. The amount of colocalization (%) is shown as box plot with inner and outlier points and mean. n(cell–cell junctions) = 15. (**f**) Sparse and dense cultures of 184A1 cells were lysed and Western blotting was performed with specific antibody against TRPV6 and E-cadherin. pAkt antibody was used to indicate the confluency of the cultures^35^. GAPDH acts as a loading control. Weight markers (kDa) for the indicated proteins are shown next to the blots. See also Supplementary Fig. S2c for uncut blots. (**g**) Quantification of the TRPV6 Western blot results, related to (**f**). Intensity values were measured in ImageJ and values for TRPV6 were divided with the corresponding GAPDH values. Control sample values were normalized to 1. Mean (± SEM) is shown. n = 3. *P < 0.05 (Paired t-test).

To explore in more detail the function of TRPV6 in the mammary epithelial cells we first performed immunofluorescence staining analyses of TRPV6 together with markers against cytoskeletal and adhesive structures. Standard immunofluorescence stainings of 4% PFA fixed 184A1 and MCF10A cells showed very diffuse staining pattern for TRPV6, while methanol-fixation removed this diffuse signal and revealed the association of TRPV6 with E-cadherin puncta at the nascent adhesions (Fig. 1a,b). Upon increasing confluency of the breast epithelial cell cultures, TRPV6 started to accumulate more at the vicinity of cell–cell junctions, partially co-localizing with E-cadherin (Figs. 1c–e; S1a,b). Besides this, immunofluorescence stainings revealed a connection with TRPV6 and intracellular cytoskeletal structures (Figs. S1c,d; S2a,b). Additionally, we observed that increasing cell density was associated with higher expression of TRPV6 (Fig. 1f,g; see also Fig. S2c). In summary, the results suggest that TRPV6 is associated with cell junction-supportive structures and that its expression and localization are influenced by mechanical tension, triggered by the neighboring cells.

### TRPV6 is essential for epithelial integrity

TRPV6 is widely expressed in various epithelial tissues, but its function and intracellular downstream pathways have remained poorly explored. As both extra- and intracellular Ca^2+^ plays a role in the maintenance of epithelial integrity, we wanted to assess the possible role of TRPV6 in the structural homeostasis of epithelial sheets.

**Figure 2:**
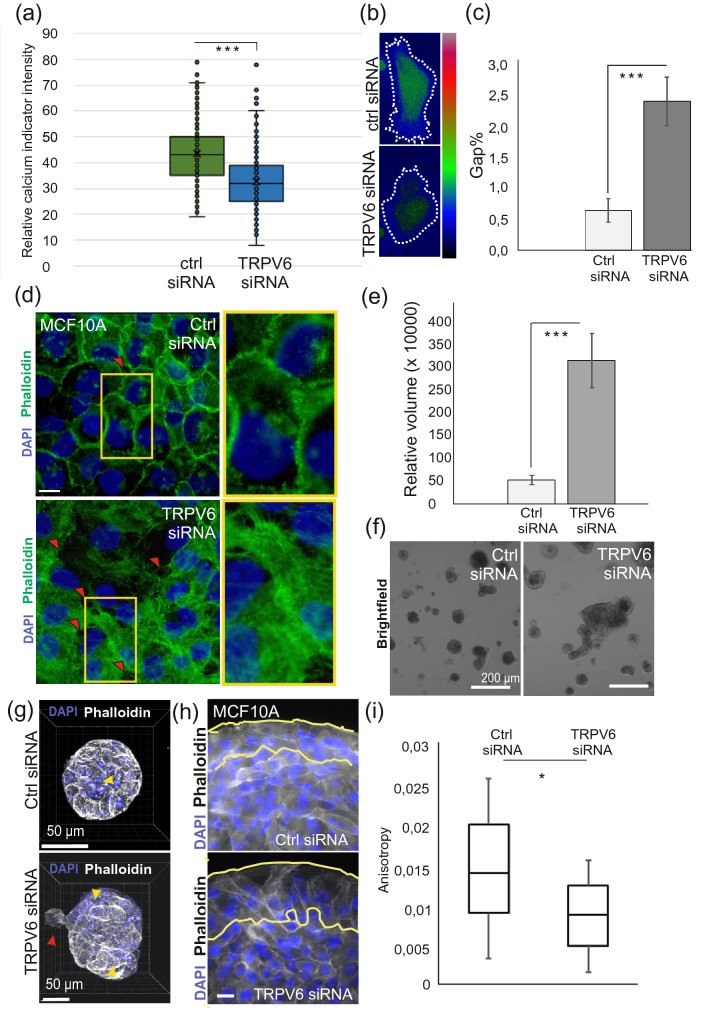
Downregulation of TRPV6 disrupts Ca^2+^-homeostasis and integrity of the mammary epithelial sheets. (**a**) Ctrl siRNA or TRPV6 siRNA-treated 184A1 breast epithelial cells were used in live cell imaging with Ca^2+^-indicator, calcium green. Calcium signal was detected with snapshots, taken from both ctrl and TRPV6-depleted cells with the same exposure time and intensity of the signal was analyzed in Fiji. Data is shown in box plots, containing inner and outlier points as well as mean. n(cells) = 300 for both ctrl- and TRPV6 siRNA. P < 0.001***, paired t-test. (**b**) Representative intensity maps for calcium green signal in ctrl- and TRPV6 siRNA cells. Cell area, used for the intensity measurements, is indicated with the dashed line. (**c**) Quantification of the percentage of gap areas in ctrl siRNA- and TRPV6 siRNA-treated MCF10A monolayers (related to **d**). Data represent mean ± SEM. n = analyzed areas from confluent monolayers; n(ctrl siRNA) = 33; n(TRPV6 siRNA) = 44; P < 0.001***, unpaired two sample Student’s t-test. (**d**) Monolayer integrity was analyzed from immunofluorescence stainings of ctrl and TRPV6-depleted MCF10A cells, where nuclei (blue) were stained with DAPI and actin (green) was stained with Phalloidin. Representative immunofluorescence (IF) images are shown in the panel and examples of gaps in between individual cells are marked with red arrowheads. Magnifications of the indicated areas are shown on the right side (yellow boxes). Bar 20 µm. (**e**) Relative volume of ctrl and TRPV6 siRNA-treated MCF10A mammo spheroids cultured in 3D matrigel for 21 days after which the samples were fixed. Data represent mean ± SEM from two individual experiments. n(ctrl siRNA) = 20; n(TRPV6 siRNA) = 18; *P *< 0.001***, unpaired two sample Student’s t-test. (**f**) Representative bright field images of ctrl and TRPV6 siRNA-treated MCF10A mammo spheroids. Bar 200 µm. (**g**) Representative images of single ctrl and TRPV6-depleted MCF10A spheroids. 3D reconstituted confocal IF images are shown with nuclei stained with DAPI (blue) and actin stained with Phalloidin (grey). Irregular lumen formation is indicated by distinct nuclear fragmentation between ctrl and TRPV6 siRNA-depleted spheroids (yellow arrow heads). Additionally, one of the cell protrusions in TRPV6 siRNA spheroid is pointed out by red arrowhead. Bar 50 µm. (**h**) Representative images of ctrl siRNA- and TRPV6 siRNA-treated MCF10A cells, grown on round collagen-coated polyacrylamide micropatterns (stiffness 4 kPa). Nuclei were stained with DAPI (blue) and actin was stained with Phalloidin (grey). Two outermost cell layers are encircled with the yellow lines, which gives the region of interest (ROI) for the analyses of anisotropy. Bar 20 µm. (**i**) Anisotropy was quantified by utilizing FibrilTool plug-in for ImageJ. n = number of monolayers; n(ctrl siRNA) = 19; n(TRPV6 siRNA) = 18; P < 0.05*; unpaired two sample Student’s t-test.

To reveal the role of TRPV6 in breast epithelium, we depleted this protein from both 184A1 and MCF10A cells by specific siRNAs (Fig. S3a–c). To verify that TRPV6 really affected the calcium homeostasis of the breast epithelial cells, we performed calcium imaging in living ctrl and TRPV6 siRNA-treated 184A1 cells (Fig. 2a,b). Loss of TRPV6 from these cells led to significantly lower signal of calcium in the utilized cell line as detected by calcium green imaging of ctrl and TRPV-depleted cells. Decreased levels of TRPV6 had also an observable impact on the epithelial integrity on both 2D and 3D cultures: on 2D cultures, loss of TRPV6 led to increased gap formation at cellular junctions (Fig. 2c,d), implicating impaired cell–cell contact formation. While in 3D cultures, mammary spheroids formed by these cells displayed abnormal morphology with higher volume, impaired sphericity and abnormal lumen formation (Figs. 2e–g; S3d,e). Additionally, decreased anisotropy of the TRPV6-depleted MCF10A cells in monolayers was detected on spherical, space-restricted micropatterns (Fig. 2h,i).

As TRPV6 was connected to E-cadherin at adherens junctions (Figs. 1a–e; S1a,b), we studied whether depletion of TRPV6 could affect this cell junction protein. While TRPV6-depleted MCF10A cells displayed less mature junctions, as visualized by E-cadherin staining (Fig. S3f), the levels of E-cadherin remained the same as detected by Western blotting from the cellular lysates of corresponding control and TRPV6 siRNA-treated cells (Fig. S4a, upper panel). In addition, the levels of tight junction protein ZO-1 remained unchanged (Fig. S4a, lower panel). Furthermore, as disruption of intact cell–cell contacts causes changes in the localization of many contact-sensitive proteins, we analyzed how compromised epithelial integrity upon loss of TRPV6 was associated with the nuclear transport of transcription factor YAP. In line with the known contact-sensitivity of this protein^36^, YAP was also found to be more nuclear in TRPV6-depleted confluent cell cultures (Fig. S4b,c). The results indicate that TRPV6 is essential for the maintenance of intact epithelial cell–cell junctions, at least in the utilized breast epithelial model system.

### TRPV6 regulates contractility of actomyosin bundles

**Figure 3:**
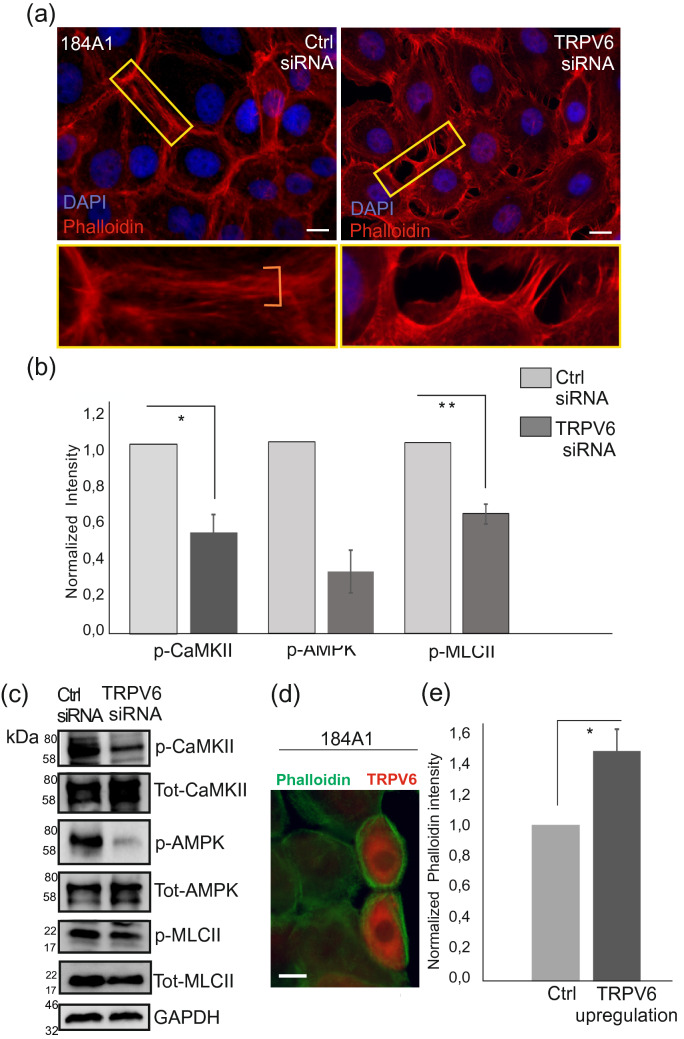
Depletion of TRPV6 affects pathways upstream of actomyosin assembly. (**a**) Human breast epithelial 184A1 cells were treated with ctrl and TRPV6 siRNAs and cultured to confluency. Immunofluorescence microscopy was utilized to detect actin-based structures, which were visualized by phalloidin staining (red). Nuclei were stained with DAPI (blue). Magnifications of the indicated cell–cell junction regions (yellow boxes) are shown on lower panels. Bars 20 µm. (**b**) Western blotting was utilized to detect levels of p-CaMKII, p-AMPK and p-MLCII from cellular lysates of ctrl and TRPV6-depleted 184A1 cells. Quantification of the intensity values were measure in ImageJ. Values for p-CaMKII, p-AMPK and p-MLCII were divided with the corresponding GAPDH values. Control sample values were normalized to 1. Mean (± SEM) is shown. Quantification of the results n(p-CaMKII) = 5; n(p-AMPK) = 4; n(p-MLCII) = 4; *P < 0.05; **P < 0.01 (Paired t-test). (**c**) Representative Western blots, related to (**b**) quantifications. Both phosphorylated forms and total levels of CaMKII, AMPK and MLCII proteins are shown. GAPDH acts as a loading control. Weight markers (kDa) for the indicated proteins are shown next to the blots. See also Supplementary Fig. 5b for uncut blots. (**d**) Exogenous expression of TRPV6-CFP in 184A1 breast epithelial cell cultures. Merged images of TRPV6-overexpressing cells (orange colour) and phalloidin (green colour) are shown. Bar 20 µm. (**e**) Intensity of the filamentous actin was quantified from the neighboring ctrl and TRPV6-overexpressing cells in ImageJ. n(cells) = 12 for both ctrl and TRPV6 overexpression, three actin filament regions were analyzed from each cell, n(fibers) = 36. Mean (± SEM) is shown. Ctrl values were normalized to 1. *P < 0.05 (Paired t-test).

We previously showed that peripheral actomyosin bundles are important in maintaining epithelial integrity through Ca^2+^ signaling^37^. As TRPV6 binds calmodulin in a Ca^2+^-dependent manner^38,39^ and Ca^2+^/calmodulin impacts actomyosin contractility at least through myosin light chain kinase (MLCK)^40^, we wanted to assess the possible role of TRPV6 in the regulation of peripheral actomyosin bundles. For this, the organization of actin cytoskeleton was analyzed in more detail with immunofluorescence microscopy from control and TRPV6 siRNA-treated 184A1 breast epithelial cells. As detected earlier, the integrity of the mammary epithelial monolayers was deficient upon TRPV6-depletion and visualization of actin with phalloidin staining revealed loss of peripheral actomyosin bundles at the cell–cell contacts (Fig. 3a). Instead, the junctions displayed sparse actin-based protrusions, making perpendicular connections to the neighboring cells (Fig. 3a, right panel; Fig. S5a, lower panel).

To further investigate whether actomyosin contractility could be affected, we utilized Western blotting and cellular lysates of TRPV6-depleted 184A1 cells. Specific antibodies against phosphorylated myosin light chain at Ser18/Thr19 (hereafter referred as p-MLCII) and phosphorylated calcium calmodulin-dependent kinase II, CaMKII, at Thr286 (hereafter referred as p-CaMKII) showed significantly decreased levels of these phosphorylated proteins (Fig. 3b,c; see also Fig. S5b), indicating that loss of TRPV6 affects actomyosin structures through the phosphorylation of MLCII. Additionally, we detected decrease in p-Thr172-AMPK (Fig. 3b,c; see also Fig S5b), a downstream target of another CaM kinase, CaMKKII, that was previously linked to the maturation of actomyosin bundles^12,37,41^.

In similar manner to TRPV6-depletion, general inactivation of mechanosensitive Ca^2+^-channels by GsMTX-4 compound in MCF10A cells led to loss of thick actomyosin structures in epithelial cell clusters (Fig. S6a). In contrast to TRPV6 interference, exogenous expression of TRPV6 led to the formation of thicker actin-based structures in 184A1 cells and concomitant increase in both p-CaMKII and p-MLCII (Figs. 3d,e; S6b,c). In all, these data indicate that TRPV6 has an important role in the dynamics of actomyosin bundles possibly through the Ca^2+^-dependent kinases, regulating phosphorylation and contractility of the actin-based structures.

### TRPV6 may potentiate invasion by tuning the expression of EMT-associated markers

TRPV6 is overexpressed in several cancers of epithelial origin and high TRPV6 expression has been detected both in invasive regions of carcinoma samples as well as in established cancer cell lines^18,22,42^. However, whether high TRPV6 itself impacts invasive progression, is not well understood.

**Figure 4:**
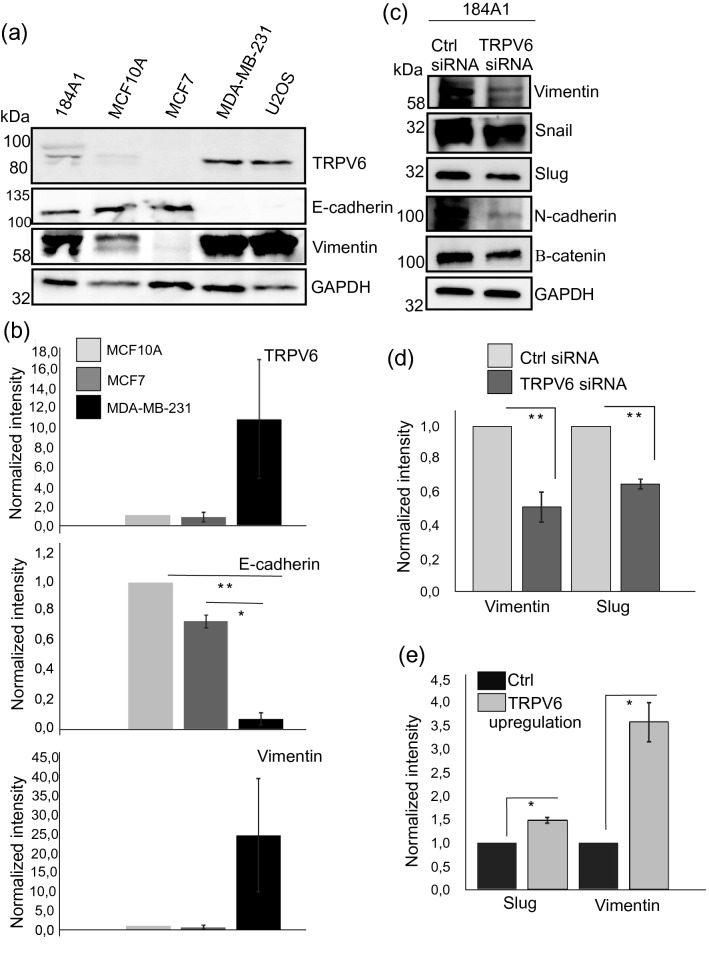
TRPV6 controls levels of EMT-associated factors. (**a**) Expression of TRPV6 in different cell lines was analyzed by Western blotting from cellular lysates, with specific antibodies. E-cadherin and vimentin were used to assess epithelial/mesenchymal-like phenotype of the cells and GAPDH was used as a loading ctrl. Weight markers (kDa) for the indicated proteins are shown next to the blots. See also Supplementary Fig. 7a for uncut blots. (**b**) Quantifications of the protein levels, related to (**a**). Levels of TRPV6, E-cadherin and vimentin were assessed from the Western blot experiments of MCF10A, MCF7 and MDA-MB-231 cell lines. Both normal mammary epithelial cells and non-invasive MCF7 mammary epithelial cells displayed low TRPV6 expression together with low vimentin and high E-cadherin expression, while invasive MDA-MB231 cells displayed totally opposite expression pattern. Mean (± SEM) is shown. n = 3. *P < 0.05; **P < 0.01 (Paired t-test). Note that although MDA-MB-231 cells displayed much higher levels of TRPV6 and vimentin, due to the variation in between individual Western blot experiments, the statistical difference in between MDA-MB-231 cells and MCF10A or MCF7 cells was not significant (Paired t-test). (**c**) Western blotting was applied to analyze the expression of EMT-associated proteins from ctrl and TRPV6-depleted 184A1 cells. Specific antibodies against vimentin, snail, slug, N-cadherin and β-catenin were utilized. GAPDH acts as a loading control. Weight markers (kDa) for the indicated proteins are shown next to the blots. See also Supplementary Fig. 7b,c for uncut blots. (**d**) Quantification of vimentin and slug levels, related to (**c**). Intensity values were measured in ImageJ and intensity values for vimentin and slug were divided with the corresponding GAPDH values. Control sample values were normalized to 1. Mean (± SEM) is shown. n = 3. *P < 0.05 (Paired t-test). (**e**) Quantification of vimentin and slug levels upon TRPV6 overexpression, related to Supplementary Fig. S7d. Intensity values were measured in ImageJ and intensity values for vimentin and slug were divided with the corresponding GAPDH values. Control sample values were normalized to 1. Mean (± SEM) is shown. n = 3. *P < 0.05 (Paired t-test).

As induction of epithelial mesenchymal transition, EMT, has been linked to calcium signaling^43,44^, we wanted to test whether expression of TRPV6 channel could impact cancer progression through regulation of EMT. For this, we analyzed TRPV6 levels from several commonly used cell lines (Fig. 4a,b; See also Fig. S7a). High TRPV6 expression seemed to correlate with the high expression of vimentin but it was inversely correlated with the E-cadherin levels. To further study the impact of TRPV6 on EMT, we assessed the levels of vimentin and other EMT-associated markers from TRPV6-silenced and -overexpressing cells by specific antibodies in “Western blotting” (Figs. 4c–e; S7b–d): Vimentin, snail, slug, *N*-cadherin and β-catenin were downregulated in TRPV6-depleted cells (Fig. 4c,d; See also Fig. S7b,c). In contrast, exogenous expression of TRPV6, led to higher levels of vimentin and slug (Figs. 4e; S7d), suggesting that TRPV6-mediated calcium influx is directly linked to the levels of EMT-markers and could in this way play a role in cancer progression.

To further understand the role of TRPV6 in mesenchymal cancer invasion, we utilized a commonly used, invasive breast carcinoma cell line, MDA-MB-231 for TRPV6-silencing. MDA-MB-231 cells display relatively high TRPV6 levels in comparison to normal breast epithelial cells and non-invasive breast carcinoma cells, MCF7. Additionally, these cells display high levels of vimentin and no detectable E-cadherin (Fig. 4a,b). Depletion of TRPV6 decreased the levels of several EMT-markers in this cancer cell line, as detected by Western blot analyses (Fig. S8). Interestingly, downregulation of vimentin was not detected in MDA-MB-231 cells upon TRPV6-depletion, while it affected the phosphorylation status of vimentin on Ser39. This specific phosphorylation site has been linked to enhanced cell migration and survival of cancer cells^45,46^. Additionally, we analyzed the impact of TRPV6-depletion on the invasion ability of MDA-MB-231 cells in circular invasion assays (Fig. S9). Surprisingly, the initial motility of TRPV6-depleted MDA-MB231 cells was higher than in control cells during this two-day experiment (Fig. S9). In conclusion, TRPV6 expression seems to determine the levels of many common EMT-linked markers in mammary epithelial cells, while the impact of TRPV6 on cancer invasion may need further studies with different types of long-term 3D-invasion assays.

## Discussion

**Figure 5:**
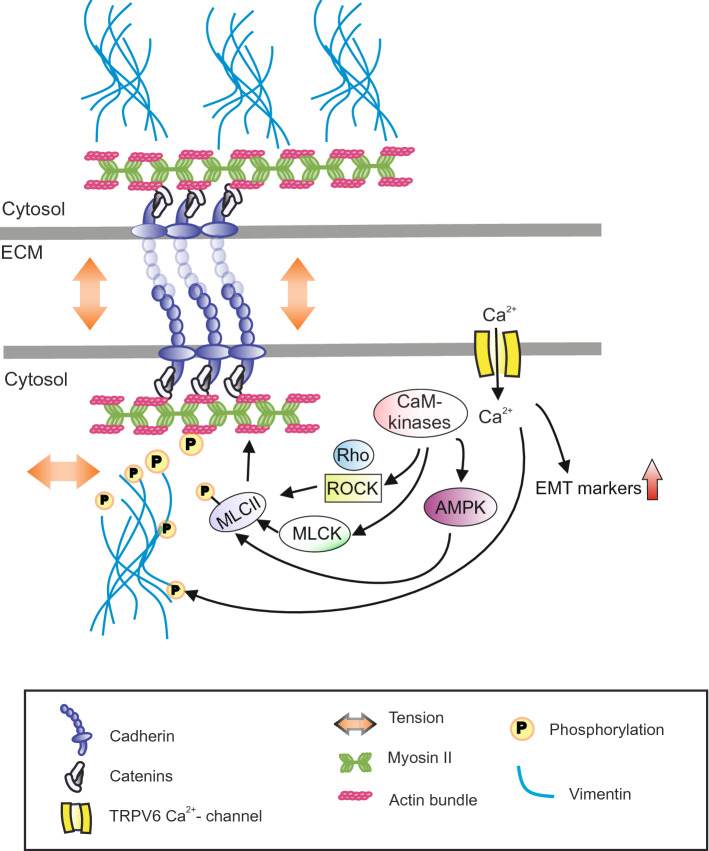
Hypothetical model. Ca^2+^-influx through TRPV6 channels controls peripheral actomyosin bundles and integrity of epithelial sheets through mediating phosphorylation of myosin light chain kinase, MLCII. This could take place through Ca^2+/^Calmodulin (CaM)-dependent kinases, CaMKII and CaMKKII as phosphorylation of CaMKII as well as phosphorylation of AMPK, a downstream target of CaMKKII, were affected by TRPV6 depletion. CaMKII has been linked to the activities of both MLCK and RhoA/ROCK pathway that direct MLCII phosphorylation^40,47–49^. Additionally, AMPK has been linked to the phosphorylation status of MLCII^12,37^. Besides controlling maintenance and contractility of actomyosin bundles though MLCII phosphorylation, TRPV6 levels were directly associated with the phosphorylation of vimentin, related to cell motility, as well as the expression levels of several EMT-linked factors that could possibly impact mesenchymal migration of the cells. One possible link in between TRPV6 and EMT markers could be EGFR/PI3K/AKT signaling pathway that is also downstream of other Ca^2+^-influx channels (reviewed in^44^). TRPV6 thus controls epithelial integrity through at least two parallel pathways.

Strictly regulated calcium homeostasis is crucial for all vital functions and abnormal Ca^2+^-influx is associated with several common pathophysiological conditions^43,44^. As TRPV6 is widely expressed in Ca^2+^-absorbing epithelial tissues, it must have an essential role in the overall maintenance of the epithelial homeostasis. In this study, we aimed in deeper understanding on the role of TRPV6 in mammary epithelial homeostasis and cancer progression. We assessed the role of TRPV6 on epithelial integrity and additionally the association of TRPV6 with the regulation of EMT markers, linked to invasive progression. Our main findings are summarized in the suggested model, Fig. 5.

TRPV6 calcium-channels are formed by four homologous TRPV6 subunits that each have six transmembrane regions^17^. In our studies we found that in the sparse cell cultures, TRPV6 protein is mainly diffuse but interestingly, also partially colocalize with cytoskeletal structures (Figs. S1 and S2). As cell density increased, the expression of TRPV6 was concomitantly upregulated with more prominent localization to the forming cell–cell junctions (Figs. 1 and S1), suggesting that its expression levels and localization are regulated in a tension-sensitive manner. Previously, TRPV6 has been shown to be translocated to the plasma membrane via Ca^2+^/Annexin I/S100A11-signaling^50^. TRPV6 was also linked to the E-cadherin based cell–cell contacts and the underlying cytoskeletal network, suggesting that it may have a role in the regulation of junctional dynamics through calcium influx. Supporting this, depletion of TRPV6 led to significantly lower calcium levels and disrupted junctional integrity, as detected both in 2D mammary epithelial sheets and as abnormal mammo sphere morphology in 3D matrigel cultures (Figs. 2, S3 and S5a). In line with these observations, excision of the *Trpv6* gene from mice results in Ca^2+^ precipitates within the enlarged, abnormal prostatic ducts and a concomitant absence of luminal infoldings^51^. TRPV6 is thus important for the maintenance of normal epithelial structures.

To better understand the role of TRPV6 in the maintenance of epithelial sheets on 2D and lobular/tubular structures in 3D, we investigated whether it could regulate the cytoskeletal structures, supporting the cell–cell contacts. Ca^2+^-signaling is known to play a major role in the maintenance of both adhesive structures and contractility of junction-supporting actomyosin bundles through phosphorylation of myosin II^52^. Several TRP family channels are also linked to cytoskeletal network and have been implicated in the dynamic changes of actin cytoskeleton during cell movement^14^. These studies have been mostly performed on the association of Canonical TRPs (TRPCs) with cell cytoskeleton, while the link in between other TRP subtypes and cytoskeletal structures have been less explored. Our study showed that TRPV6 indeed associates with cytoskeletal structures and can regulate actin dynamics: Decreased TRPV6 led to disruption of the normal peripheral actomyosin bundles, supporting the integrity of the epithelial sheets (Fig. 3). Loss of actomyosin bundles could take place due to impaired phosphorylation of MLCII through altered CaM-dependent kinase activities in TRPV6-deficient cells. While TRPV6 seems to support the actomyosin network, cytoskeletal structures themselves may also provide physical support for the membrane-embedded TRP proteins, making this relationship bidirectional.

TRPV6 is linked to poor clinical outcome in several cancer types^22,27,50^ and elevated Ca^2+^-signaling has been linked to numerous pathological conditions and progression of invasive cancers^44,53^. Related to cancer invasion, abnormal Ca^2+^ homeostasis has been associated with epithelial mesenchymal transition and we also detected an interdepency in between TRPV6 expression and the levels of many EMT-associated factors (Figs. 4; S7 and S8)*.* Physical changes in the tumor microenvironment have been suggested to play a major role in the invasive progression. However, not much is known about the mechanisms that bridge the microenvironment and nuclear transcription factors to induce EMT-like phenotype. In this study we show that TRPV6 is expressed in a tension-dependent manner, higher cell-density being linked to the higher expression of this Ca^2+^-channel protein. Upon cancer progression, abnormal proliferation and elevated stiffness of the stroma could thus hypothetically induce expression of TRPV6, leading to upregulation of EMT markers. EMT is often the initial step in tumor spreading and expression of EMT markers have been associated with both basal- and claudin-low breast carcinoma subtypes that have poor prognosis^54–56^. In line with these previous findings and our studies, also high expression of the melastatin-related transient receptor potential 7 and 8 (TRPM7 and TRPM8) proteins have been linked to the expression of EMT markers and invasive ovarian and breast cancers^57–59^. These studies showed that the connection in between TRPM-channels and EMT markers was dependent on PI3K/AKT signaling and this could possibly play a role in TRPV6-induced EMT as well. Besides TRPM7 and 8, also other Ca^2+^-influx channels within the TRP-family and other Ca^2+^-channel protein families have been associated with progression of various types of cancers through EMT and chelation of free cytosolic Ca^2+^ suppresses the expression of several mesenchymal markers in breast, hepatic and colon cancer cells (reviewed in Iamshanova et al.^44^). To test whether overexpression of TRPV6 could induce EMT-dependent cancer cell migration, we performed circular invasion assays with MDA-MB-231 cells (Fig. S9). In these assays, we surprisingly observed slightly higher motility of the TRPV6 siRNA cells in comparison to the control MDA-MB-231 cells. Therefore, further studies with long-term 3D culture setups may be required to verify whether TRPV6-linked changes in EMT markers impact invasion process. Interestingly, research by Dhennin-Duthille et al.^22^ showed that depletion of TRPV6 from MDA-MB-231 cells inhibits invasion, but the mechanisms behind this were not understood.

Besides affecting EMT-associated factors, TRPV6 expression was closely related to the phosphorylation status of CaMKII (Figs. 3 and S5b). Activity of CaMKII has been linked to the regulation of actomyosin structures: phosphorylated, active CaMKII has been shown to downregulate the activity of MLCK in smooth muscle cells and therefore lower the contractility of actomyosin structures^47, 48^. On the other hand, CaMKII has also been linked to the activation of Rho-ROCK pathway and could therefore also induce contractility^49^. In our studies, TRPV6-depletion-associated with decrease in p-CaMKII and decrease in p-MLCII (Fig. 3). Whether this decreased in p-MLCII was due to lower CaMKII activity or due to decreased activity of CaMKKII, upstream of AMPK, needs further studies. In addition to the regulation of actin-based structures, higher CaMKII levels directly correlate with the potential to invade through induction of EMT in breast cancer models^60^. Therefore, TRPV6-linked changes in EMT-associated factors, could also take place due to altered phosphorylation status of CaMKII. CaMKII has been linked to the regulation of EGFR through phosphorylation of its cytoplasmic tail^61^ and downstream pathways of EGFR signaling regulate the expression of several EMT-associated markers^62^. TRPV6 could thus impact invasive progression through EGFR pathway. Also, TRPM7 channel-mediated Ca^2+^-influx has been implicated in EMT through the regulation of EGF-receptor^57^.

Taken together, any distortions in the regulation of TRPV6 channel, leading to either higher or lower TRPV6 levels, can impair the ability of the cells to properly respond to extracellular tension and lead to pathophysiological conditions: too low levels of TRPV6 affect the integrity of the epithelium through impaired maintenance of cell–cell junctions, while high TRPV6 leads to overexpression of EMT markers. In the future, it would thus be important to study in more detail how biophysical changes in the tissue microenvironment can trigger TRPV6 and cause changes in the intracellular signaling cascades. As TRPV6 was expressed in a tension sensitive manner, one could assume that stiffening environment along cancer progression, plays a role in the high TRPV6 expression. Knowing the early events that trigger EMT in cancer cells may help in identifying therapeutic targets to control cancer cell invasion and prevent metastasis. A number of TRPV6 inhibitors have already shown potential in reducing cell growth f.i. in breast cancer models^63–67^. Whether these same inhibitors also impact EMT, needs to be studied. Additionally, highly homologous TRPV6 and TRPV5 as well as other TRP-family channel proteins have been reported to hetero-oligomerize^50,68,69^, and the interplay and possible compensatory expression mechanisms of these proteins should be assessed in the future.

## Methods

### Cell culture

Human breast epithelial cells, MCF-10A (ATCC, CRL-10317) and 184A1 (ATCC, CRL-8798), were cultured in DMEM/F12 (Invitrogen) with 5% horse serum, 20 ng/ml EGF, 0.5 µg/ml Hydrocortisone, 100 ng/ml Cholera Toxin, 10 µg/ml Insulin and Pen/Strep. MDA-MB231 cells were cultured in DMEM/F12 media with 10% fetal bovine serum and Pen/Strep. Cells were grown at 37 °C in 5% CO_2_ and subcultured every 3–4 days. Cells were transfected with 25–50 nM siRNAs for human TRPV6 (#55503, Dharmacon, target sequence GGAAACAGCGCUACACAUA) by using RiboJuice siRNA Transfection Reagent (#71115, Novagen) according to manufacturer’s protocol. A scrambled, non-targeting negative control was used in control plates (AllStars negative control siRNA, QIAGEN). Transfected cells were incubated 72 h, retransfected if needed and proceed to immunofluorescence stainings or western blotting. The efficiency of RNAi was determined by Western blot.

### 3D cultures

For 3D cultures, 50 µl/well of GFR Matrigel (#356230, Lot. 1,272,006 and 6,172,006, Corning) was added into pre-chilled 8-well chamber slide (Lab-Tek Chamber Slide with cover glass slide, Thermo-Fisher Scientific) using pre-chilled tips. ~ 5,000 cells in F12 media were seeded on top of Matrigel-coated chamber slides and incubated for 22 days. During this time cells were transfected three times with TRPV6 siRNAs as with the 2D cultures. Transfections were done on day 4th, 8th and 19th. Medium renewal (F12 medium + 4% Matrigel) was done in every 3–4 days and after 22 days cultures were fixed and stained for immunofluorescence microscopy.

### Immunofluorescence stainings

For 2D cell cultures, cells were plated on laminin (50 µg/ml, #L2020, Sigma-Aldrich) coated coverslips. Fixation was done with 4% PFA at room temperature for 20 min. After 5 min of permeabilization with 0.1% Triton X-100 in PBS, cells were washed with Dulbecco-0.2% BSA. Alternatively, ice cold methanol was used for fixation after washing cells with cold PBS. Incubation in methanol was done at − 18 °C for 10 min. Primary antibodies were incubated for 1 h and secondary antibodies and Phalloidin were incubated for 30–45 min. The following primary antibodies were used in 1:50 dilutions: TRPV6 α-rabbit antibody (#SAB2106366, Sigma-Aldrich), E-cadherin α-mouse (#14472S, CST) or α-rabbit E-cadherin (#3195, CST), α-rabbit YAP, 1:50 (#14074, CST), α-mouse CK5 (ab17130, Abcam). Alexa Fluor Phalloidin 488 or 647 (#A12379, #A22287, respectively, Thermo Fisher Scientific) were used for staining of actin cytoskeleton in 1:300 dilution. Nuclei were stained with DAPI (1:3,000 dilution, Thermo Fisher Scientific) and samples were mounted with Mowiol-Dabco. 2D cultures were imaged with Leica DM6000B using 20×/0.7 HC PL APO CS wd = 0.59, 40×/1.25–0.75 HCX PL APO CS Oil wd = 0.10 and 63×/1.40–0.60 HCX PL APO Lbd.bl. Oil wd = 0.10 objectives.

Cells on matrigel were washed with PBS and fixed for 20 min with 2% PFA. Permeabilization was done for 10 min with 0.25% Triton X-100 in PBS. Cultures were blocked for 1 h at RT in IF buffer (0.1% BSA, 0.2% Triton x-100 and 0.05% Tween in PBS) + 10% goat serum. Primary antibodies were incubated at + 4 °C o/n. Secondary antibody and phalloidin were incubated for 45 min following 10–15 min DAPI staining. Mounting was done with Mowiol-Dabco. 3D samples were imagined with Leica TCS SP5 using HCX PL APO 20×/0.7 Imm Corr (water, glycerol, oil) Lbd.bl objective.

### Western blotting

Transfected cells were washed with cold PBS and lysed for 20 min on ice with 1% Triton X-100 with protease and phosphatase inhibitors Protease inhibitor Cocktail Set III (#539134, Calbiochem), Phosphatase Inhibitor Cocktail Set II (#524625, Calbiochem). Subsequently cells were scraped from the plates following 12,000 rpm centrifugation at + 4 °C for 20 min. Prior to SDS-PAGE (Mini-PROTEAN TGX, Precast gels, 4–15%, #456-1084, Bio-Rad) samples were denatured 5–15 min at 50–75 °C depending on the immunoblotted protein in question. Used Protein Ladder was from New England Biolabs (#P77125). Proteins on the gels were transferred on PVDF membranes (#IPVH00010, Millipore) using semi-dry (#170-3940, Bio-Rad) or wet transfer systems. Membranes were blocked with 5% milk/BSA for 1 h on RT shaker. Primary antibodies were incubated o/n on 4 °C shaker and secondary antibodies for 45 min–1 h on RT shaker. Luminata Crescendo Western HRP Substrate (#WBLURO100, Millipore) was used when proteins were detected with Fujifilm LAS-3000 imager. The following primary antibodies were used in 1:1,000 dilutions: TRPV6 α-rabbit antibody (#SAB2106366, Sigma-Aldrich), α-rabbit p-AMPK (Thr172) (#2335, CST) and α-rabbit Thr18/Ser19 p-MLCII (#3674, CST), α-rabbit MLCII (#3672, CST), , α-rabbit p-CaMKII (Thr286) (#12716, CST), α-rabbit vimentin (#5741, CST), α-rabbit slug (#9585, CST), α-rabbit p-Akt (Ser4739) (#4060, CST), α-rabbit Snail (#3879, CST), α-rabbit N-cadherin (#13116, CST), α-rabbit Beta-catenin (#8480, CST) and α-rabbit GAPDH (G9545, Sigma-Aldrich). Secondary antibodies for western blotting were species-specific horse-radish peroxidase (HRP) diluted in 1:3,000–5,000 (CST).

### Ca^2+^ -imaging

Calcium imaging assay was performed with ctrl siRNA and TRPV6 siRNA-treated cells. Cells were replated on CELLview imaging dishes (CELLview, Greiner bio-one) and incubated with 1 µM cell permeant Calcium Green-reagent (Calcium Green-1, AM, Thermo Fischer Scientific; Stock in DMSO) for 1 h, after which the calcium indicator was washed away and cell culture media was replaced. Imaging was performed within two hours after washing. 3I Marianas imaging system (3I intelligent Imaging Innovations), with an inverted spinning disk confocal microscope Zeiss Axio Observer Z1 (Zeiss) and a Yokogawa CSU-X1 M1 confocal scanner was utilized. The setup has appropriate filters, heated sample chamber (+ 37 °C), and controlled CO_2_. A 20×/0.8 Plan-Apochromat Ph2 WD = 0.55 M27 objective was used. SlideBook 5.0 software (3I intelligent Imaging Innovations) and sCMOS (Andor) Neo camera were used for the image acquirement and recording. Ca^2+^-indicator signal was recorded from multiple fields containing tens of cells. Images were acquired with the same settings and exposure time for all samples. After imaging, the raw data was exported to Fiji for the analyses of Ca^2+^-indicator intensity. Cells were picked by using multi-point setting and automatically received intensity values were exported to excel for further analyses. Paired t-test was used to calculate the significance in between the two groups.

### Analysis of monolayer integrity

Immunofluorescence images from Phalloidin-stained monolayer cultures were analyzed with ImageJ. Monolayer areas with 55–67 cells were chosen for further analysis. Nuclei stained with DAPI were counted from 8-bit images with particle analysis. Images with > 1 mitotic cell were eliminated from the analysis. Images were processed to binarize cell-devoid and cell-covered areas. DAPI channel was retained in analysis to prevent nucleoli from contributing to dark areas. All images were equally processed at each step: after brightness and contrast enhancement, median filter was applied to smoothen images. Further, by adjusting threshold, binary images were produced with ImageJ tool, where empty areas between cells corresponded to a specific, quantifiable area. The equivalence between original and binary image was checked each time to ensure accuracy of the measurement. The results ultimately showcase the calculated percentage of the area devoid of cells, as in gaps between cells (gap %). Two-tailed unpaired Student’s t-test was used for statistical significance.

### Imaging data and statistical analyses

Confocal 3D stack images were reconstituted and analyzed with Bitplane Imaris suite software. Surface statistics were used to measure the sphericity, volume, and area of the spheroid. Two-tailed unpaired Student’s t-test was used to reveal statistical significance.

Actin cytoskeleton organization was analyzed with ImageJ FibrilTool plug-in^70^ from spherical monolayer cultures. The spherical shape of the monolayers was obtained by culturing ctrl and TRPV6 siRNA-depleted cells on round collagen-coated polyacrylamide micropatterns with stiffness of 4 kPa. Images of the monolayers comprise ¼ of the whole monolayer circle pattern. To analyze the organization of actin at the edges of the monolayers, two outermost cell layers were lined. Anisotropy was computed from this lined area with FibrilTool. Two-tailed unpaired Student’s t-test was used to reveal statistical significance.

ImageJ was used to quantify relative differences between Western blot protein bands. From each grayscale TIFF image mean gray value was measured for separate bands using fixed frame size. The background was subtracted from the original pixel density inverted value. Control values were normalized to 1. Data from independent experiments were normalized and reported as mean ± SEM.

Comparison of the phalloidin intensity in between control and TRPV6-overexpressing cells was performed from the same image fields, containing both several ctrl and TRPV6-overexpressing cells. Totally 4–5 Image fields were analyzed and from each field 3 control cells and 3 overexpressing cells were measured. The intensity of 3 fibers from each cell was quantified in ImageJ and the values within one image field were always normalized so that the control values were set to 1 and the values of TRPV6-overexpressing cells were normalized accordingly. All together 36–45 fibers (U2OS and 184A1 cells, respectively) were analyzed from both control and TRPV6 overexpressing cells. Statistical significance was verified by paired t-test.

Colocalization of E-cadherin and TRPV6 was analyzed in Fiji. Area of E-cadherin staining at the cell–cell junctions was used as a region of interest, ROI, and TRPV6, covering this ROI (in %) was analyzed with Fiji colocalization analysis.

### Invasion assay

Control and siRNA treated MDA-MB-231 cells were plated in 12-well plates with ibidi cell-culture insert for wound healing (ibidi, Gräfelfing, Germany) in the center of each well. The inserts were removed and the cells were overlayed with Matrigel (Sigma-Aldrich). Images were taken every 30 min for 48 h with CellIQ imaging device (CM technologies, Tampere, Finland). The area progressed from the cells was calculated based on area measurements with ImageJ software.

## Supplementary information

Supplementary Figures.

## Data Availability

This study did not generate any unique datasets or code. All raw data is available on request.
