# Supplementary material for: TRPV6 calcium channel directs homeostasis of the mammary epithelial sheets and controls epithelial mesenchymal transition
Source: Sci Rep. 2020 Sep 7;10:14683. doi: 10.1038/s41598-020-71645-z (PMC7477193; doi:10.1038/s41598-020-71645-z)
Supplement: Supplementary file 1 — Supplementary Figures. [file 41598_2020_71645_MOESM1_ESM.pdf]

SUPPLEMENTARY INFORMATION

SUPPLEMENTARY FIGURES AND SUPPLEMENTARY FIGURE LEGENDS

TRPV6 calcium channel directs homeostasis of the mammary epithelial sheets and controls epithelial mesenchymal transition

Tytti Kärki [1,2], Eeva Kaisa Rajakylä [1], Anna Acheva[1] and Sari Tojkander [1]

[1]Section of Pathology, Department of Veterinary Biosciences, University of Helsinki, Finland
[2]Department of Applied Physics, School of Science, Aalto University, Finland

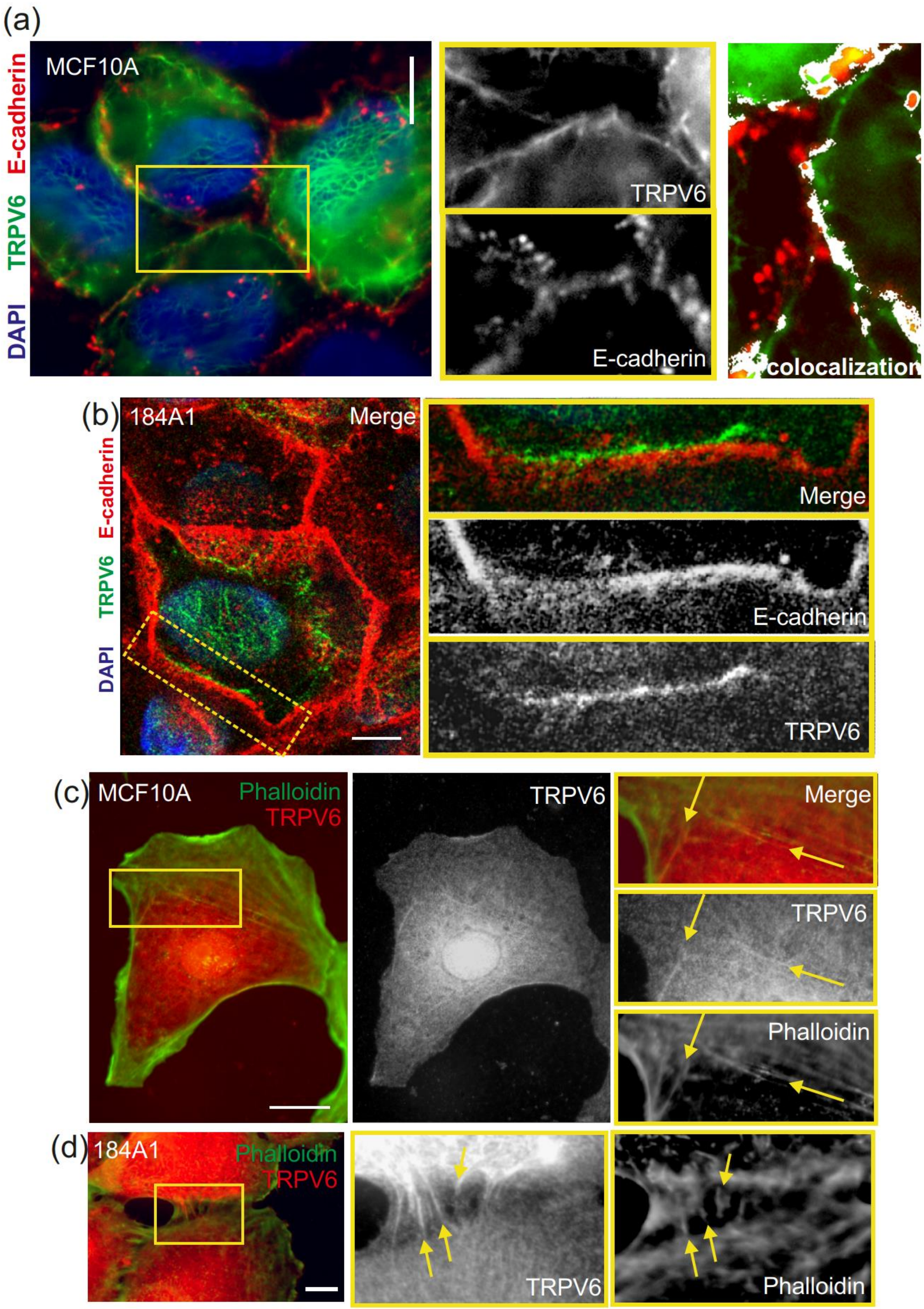

Figure S1. Localization of TRPV6. a) Methanol-fixed, confluent MCF10A cells were stained with specific antibodies against TRPV6 and E-cadherin. DAPI was used to visualize nuclei.

Magnifications from the cell-cell junction area (yellow box) are shown in the middle panel. Right panel shows colocalization as analyzed with ImageJ (white areas). Bar 10 um. **b)** Confluent 184A1 mammary epithelial cell cultures were fixed with methanol and immunofluorescence microscopy was applied to detect the localization of TRPV6 and E-cadherin. Magnification of the cell-cell junction is shown in yellow box. Nuclei were stained with DAPI. Bar 10 um. **c)** PFA-fixed MCF10A cells were stained with specific antibody against TRPV6 and phalloidin to visualize actin cytoskeleton. Magnifications of the indicated area (yellow box) is shown on the right panel. Yellow arrows point the colocalization of TRPV6 with the actin-based structures. Bar 20 um. **d)** Semi-confluent PFA-fixed 184A1 cells were stained with specific antibody against TRPV6 and phalloidin to visualize actin cytoskeleton. Magnifications of the indicated area (yellow box) is shown on the middle and right panel. Yellow arrows point the colocalization of TRPV6 with the actin-based structures at the nascent adhesions. Bar 20 um.

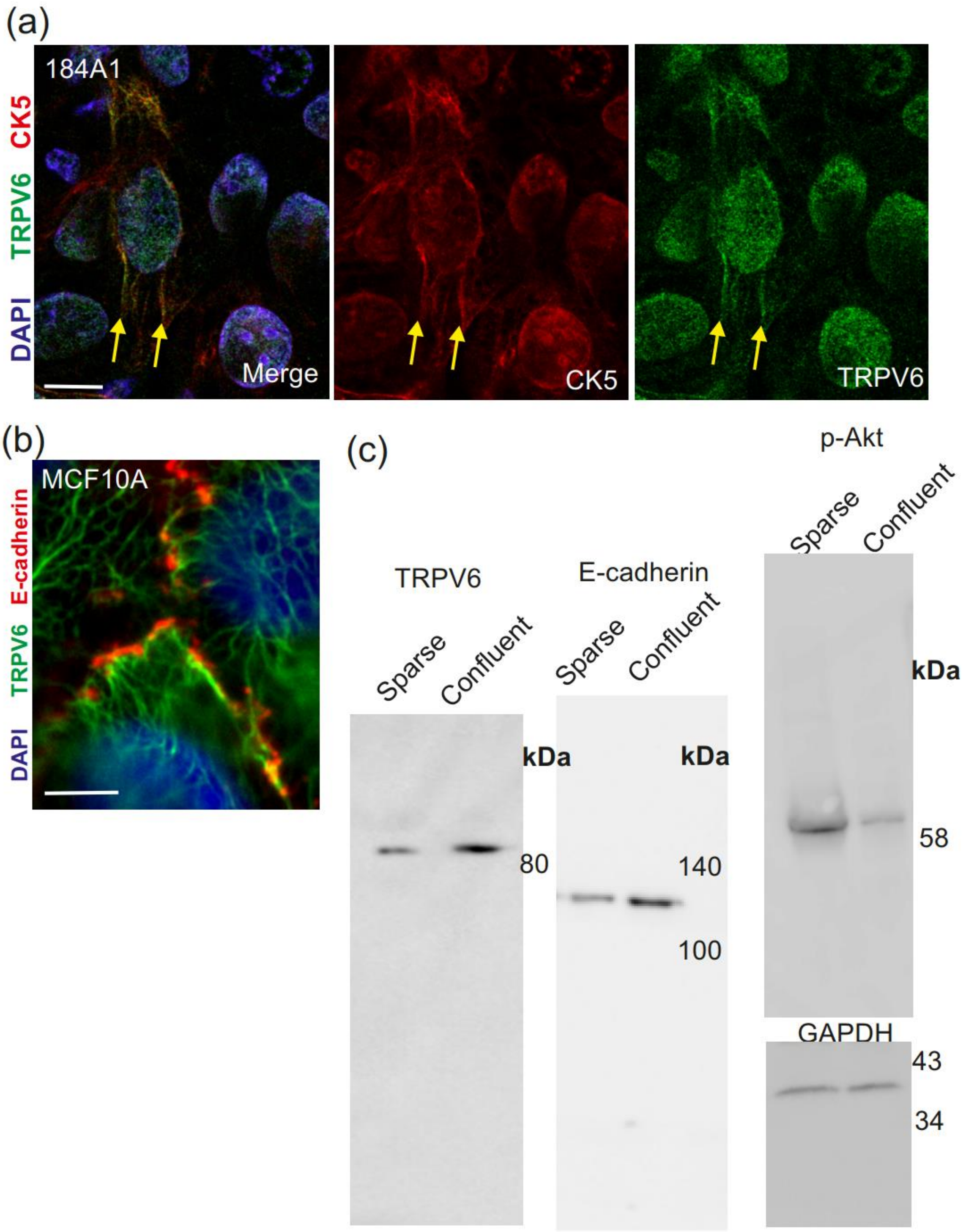

Figure S2. Localization and expression of TRPV6. a) Semi-confluent, methanol-fixed 184A1 cells were stained with specific antibodies against TRPV6 and CK5. DAPI was used to visualize nuclei. Yellow areas indicate colocalization of TRPV6 with CK5. Bar 20 um. **b)** Methanol-fixed semiconfluent MCF10A cells were stained with specific antibodies against TRPV6 and E-cadherin. Nuclei were stained with DAPI. Loss of cytoplasmic diffuse TRPV6 reveals filamentous appearance

of TRPV6. Bar 10 um. **c)** Uncropped Western blots, related to Figure 1f. Weight markers (kDa) for the indicated proteins are shown next to the blots.

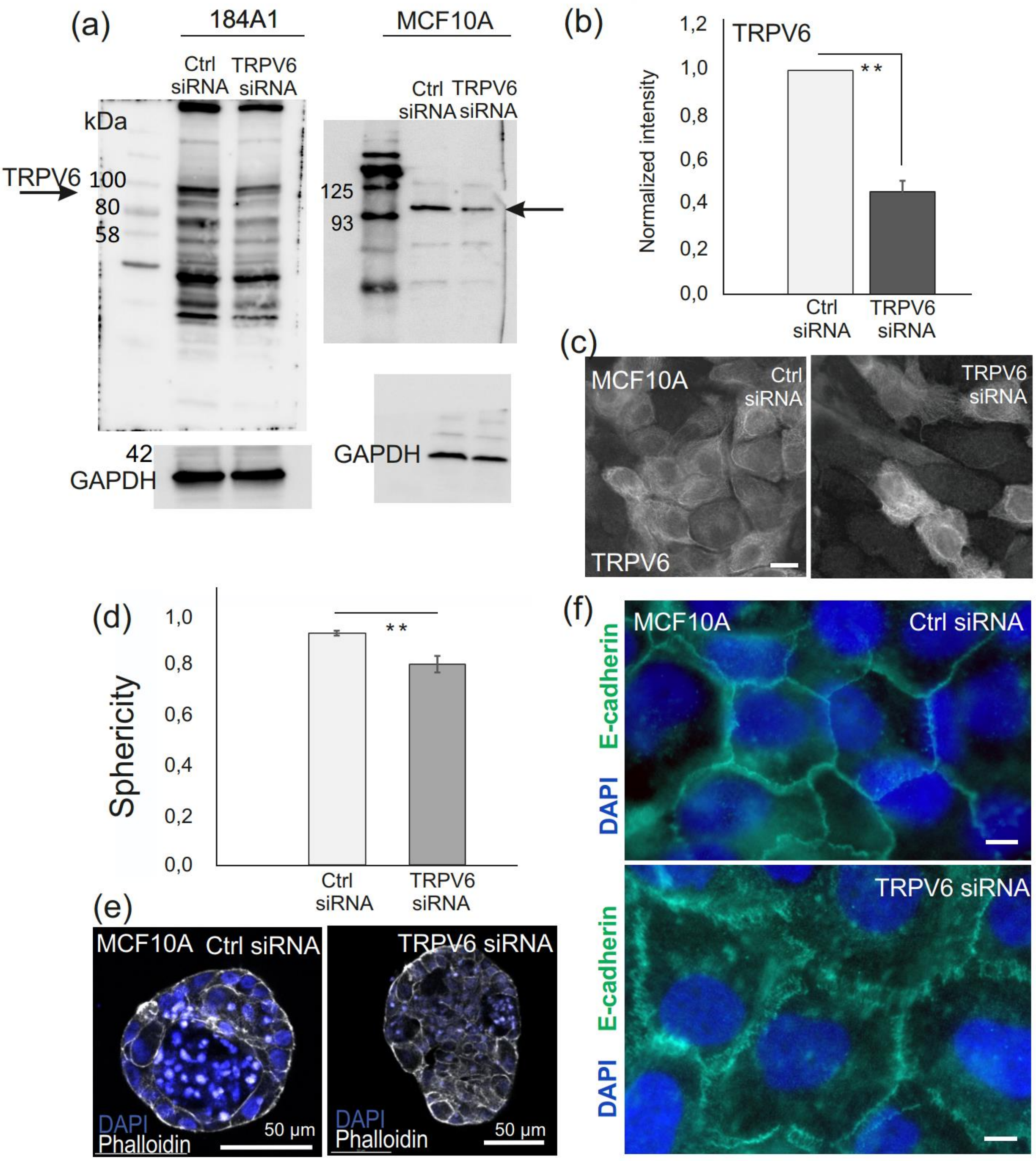

Figure S3. TRPV6 impacts integrity of the epithelial cell-cell junctions. a) Efficiency of TRPV6 siRNA in 184A1 and MCF10A breast epithelial cell lines. Ctrl and TRPV6 siRNAs were incubated for 3 days. Cellular lysates of ctrl and TRPV6-depleted cells were used in Western blotting to confirm the downregulation of TRPV6. Representative Western blots are shown. Specific antibody against

TRPV6 was used and GAPDH acts as a loading control. Weight markers (kDa) are shown next to the blots. **b)** Quantification of TRPV6 siRNA results in 184A1 cells, related to Fig. S3a. Intensity values were measure in ImageJ and intensity values for TRPV6 were divided with the corresponding GAPDH values. Control sample values were normalized to 1. Mean (+/- SEM) is shown. n= 3. **P<0.01 (Paired t-test). **c)** Representative immunofluorescence images of the TRPV6 siRNA experiments in MCF10A cells, related to Figures S3a and b. TRPV6 was detected with specific antibody, which shows diffuse pattern in PFA-fixed cells. Bar 20 um. **d)** Sphericity of the mammo spheres was quantified from fixed ctrl siRNA and TRPV6-depleted MCF10A 3D matrigel samples. Data represent mean ± SEM from two individual experiments. n(ctrl siRNA) = 20 and n(TRPV6 siRNA)= 18; *P* < 0.01 **, unpaired two sample Student's t-test. **e)** Representative images of ctrl and TRPV6 siRNA-treated MCF10A cell cultures in 3D matrigel after three weeks of culture. Phalloidin was used to visualize actin cytoskeleton and DAPI for nuclei. Bar 50 um. **f)** Confluent ctrl and TRPV6 siRNA-treated MCF10A monolayers were fixed and utilized for immunofluorescence microscopy. Specific antibody against E-cadherin was used to detect possible disruption of the cell-cell junctions. DAPI was used to visualize the nuclei. Bar 10 um.

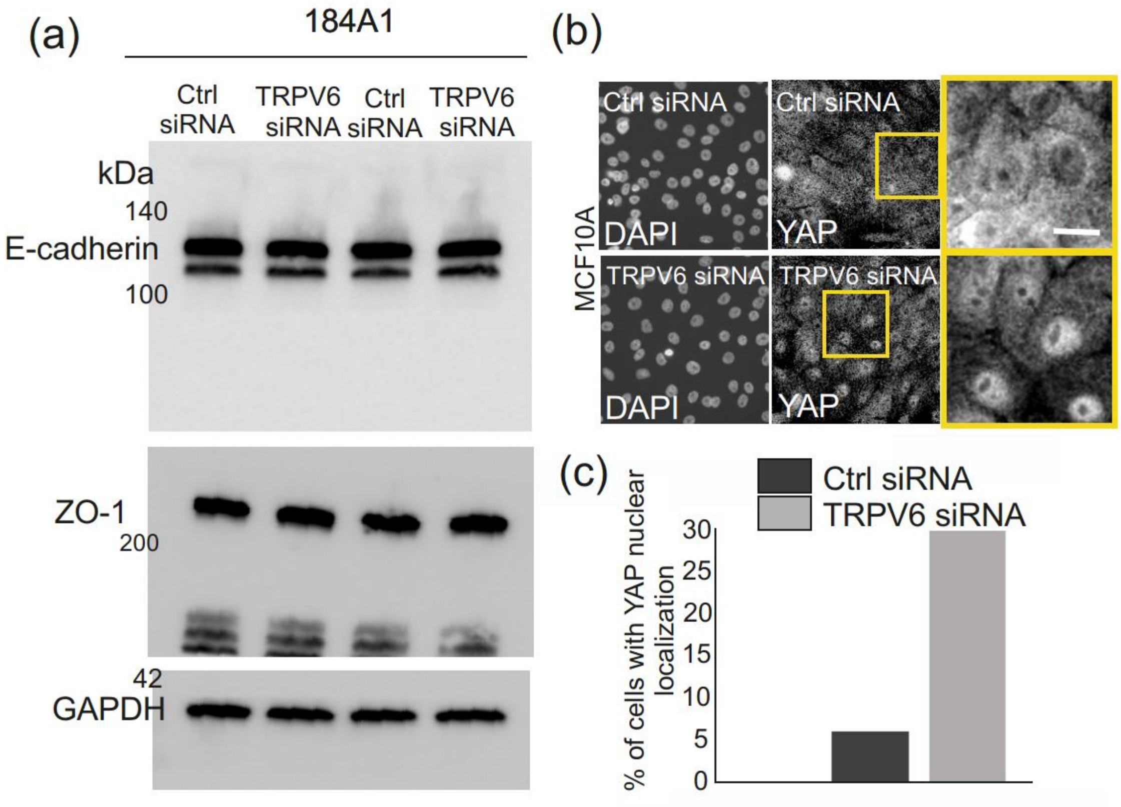

Figure S4. TRPV6-depletion leads to impaired epithelial integrity. a) Cellular lysates from TRPV6-siRNA experiments in 184A1 cells were used for Western blotting. Antibodies against E-cadherin and ZO-1 were used to assess the levels of these cell-cell junction proteins. GAPDH was used as a loading control. Weight markers (kDa) are shown next to the blots. **b)-c)** Analysis of YAP localization in TRPV6-depleted cells. Localization of YAP was analyzed from confluent cultures of MCF10A cells, treated with either control siRNA or TRPV6 siRNA for 3 days. In the analyzed control cell monolayers 27 out of 420 analyzed cells were displaying nuclear YAP localization (6%), while in TRPV6-depleted cells 93 cells out of analyzed 309 cells (30%) were having nuclear YAP. YAP was stained with a specific antibody and nuclei were visualized with DAPI. Bar 20 um.

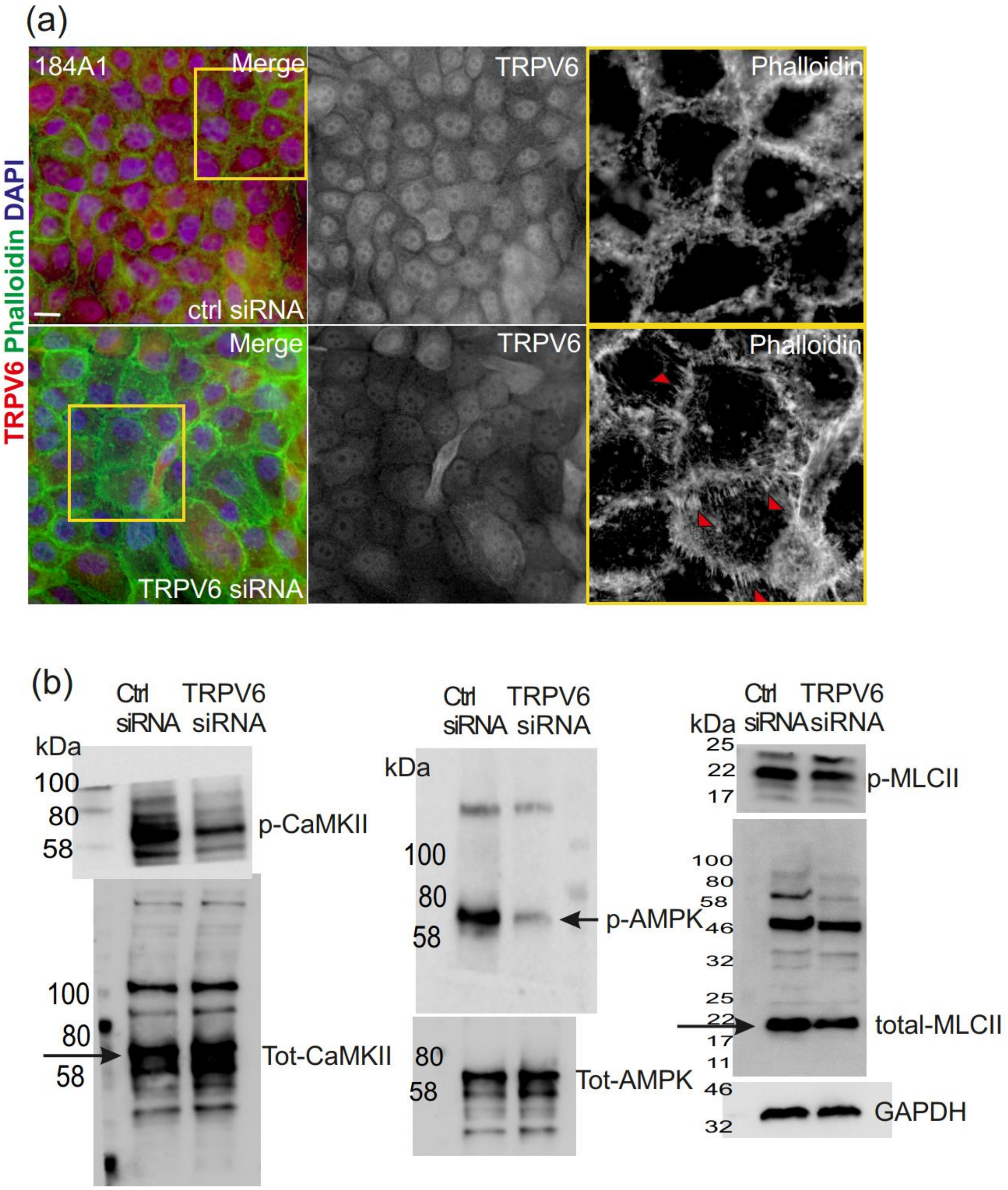

Figure S5. TRPV6 impacts epithelial integrity through actomyosin dynamics. **a)** Depletion of TRPV6 from 184A1 breast epithelial cells. Immunofluorescence stainings of ctrl siRNA and TRPV6 siRNA cells, stained with TRPV6 are shown. Actin is visualized with phalloidin and nuclei with DAPI. Magnifications of the indicated areas (yellow boxes) of phalloidin stainings are shown on the right side. Red arrows point to disrupted, spiky junctions in between TRPV6-depleted cells. Bar 20

um. **b)** Uncropped Western blots, related to Fig. 3c are shown. Weight markers (kDa) are shown next to the blots and arrows indicate the protein of interest.

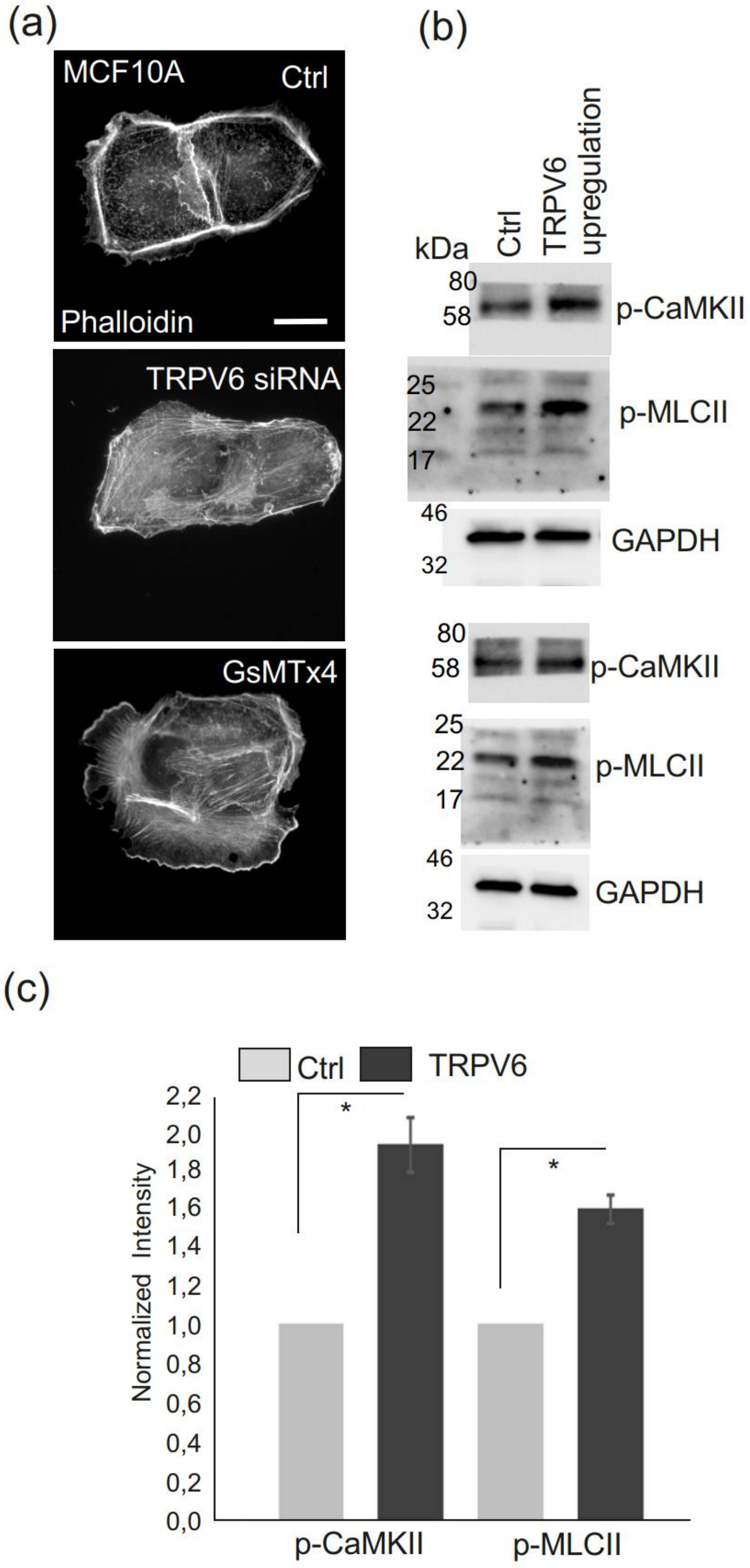

Figure S6. TRPV6 regulates actomyosin network by controlling phosphorylation of MLCII. a) Depletion of TRPV6 calcium channel or inhibition of mechanosensitive Ca^{2+}-channels by chemical compound GsMTx4 leads to disruption of thick actomyosin bundles in MCF10A cells as detected

with immunofluorescence stainings. The results indicate that Ca^{2+}-channels are important for the maintenance of actomyosin bundles in epithelial cells. Actin is visualized with phalloidin. Bar 20 um. **b)** Exogenous expression of TRPV6 in 184A1 breast epithelia cells. Western blotting was utilized to detect the levels of phosphorylated CaMKII (Thr286) and MLCII (Ser18/Thr19) from the cellular lysates. GAPDH was used as a loading control. Uncropped Western blots are presented and the weight markers (kDa) are shown next to the blots. **c)** Quantification of the Western blots, related to Fig. S6b. Intensity values were measured in ImageJ and intensity values for p-CaMKII and p-MLCII were divided with the corresponding GAPDH values. Control sample values were normalized to 1. Mean (+/- SEM) is shown. n(p-CaMKII)=3, n(p-MLCII)= 4. *$P<0.05$ (Paired t-test).

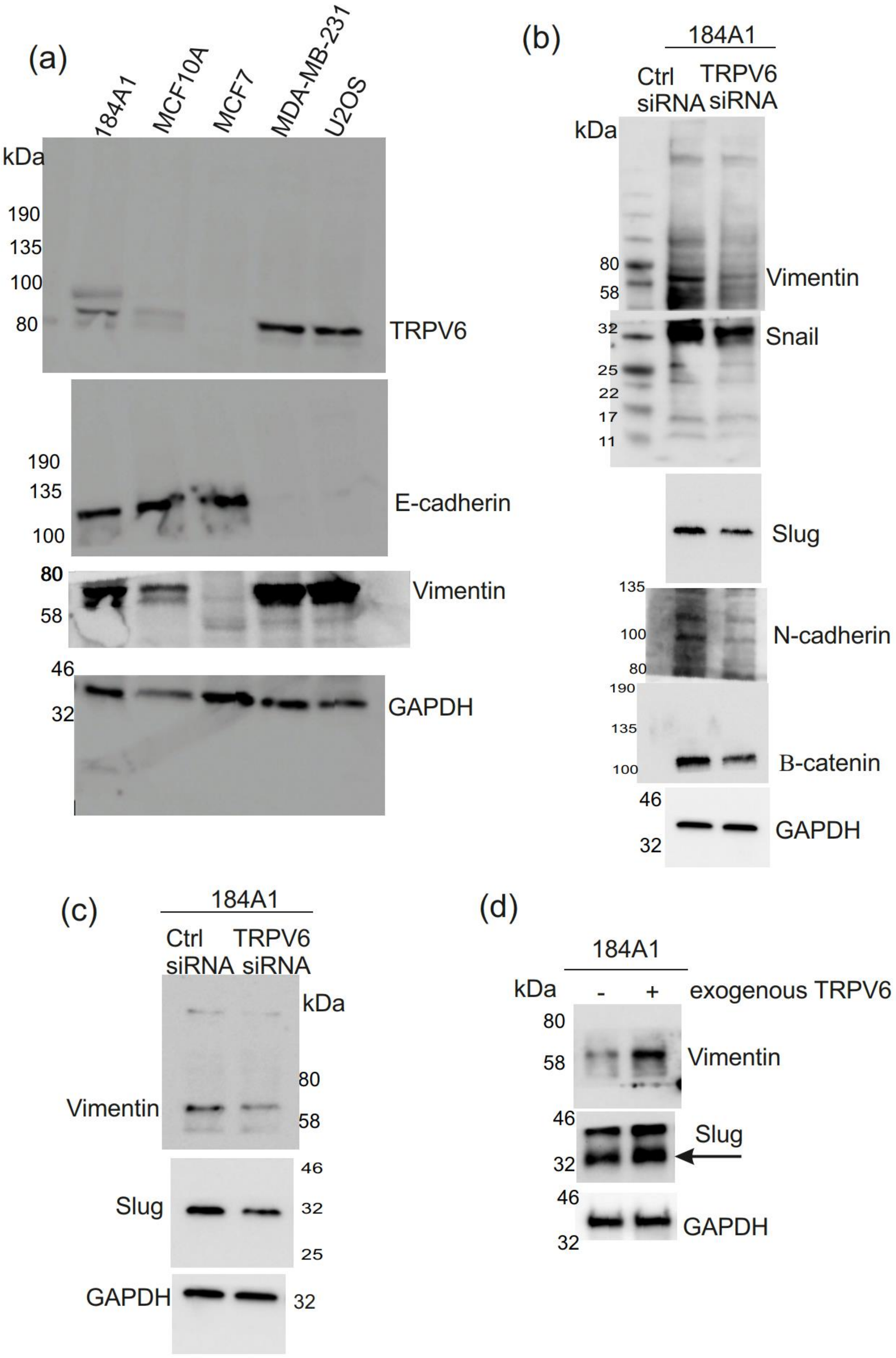

Figure S7. TRPV6 controls the expression of EMT-associated proteins. a)-c) Uncropped Western blots, related to Figure 4. Weight markers (kDa) for the indicated proteins are shown next to the blots.

d) Exogenous expression of TRPV6 in 184A1 breast epithelial cells is associated with increased levels of EMT-linked factors. Examples on vimentin and slug are shown. GAPDH acts as a loading control. Corresponding quantification of the results is shown in Fig. 4e.

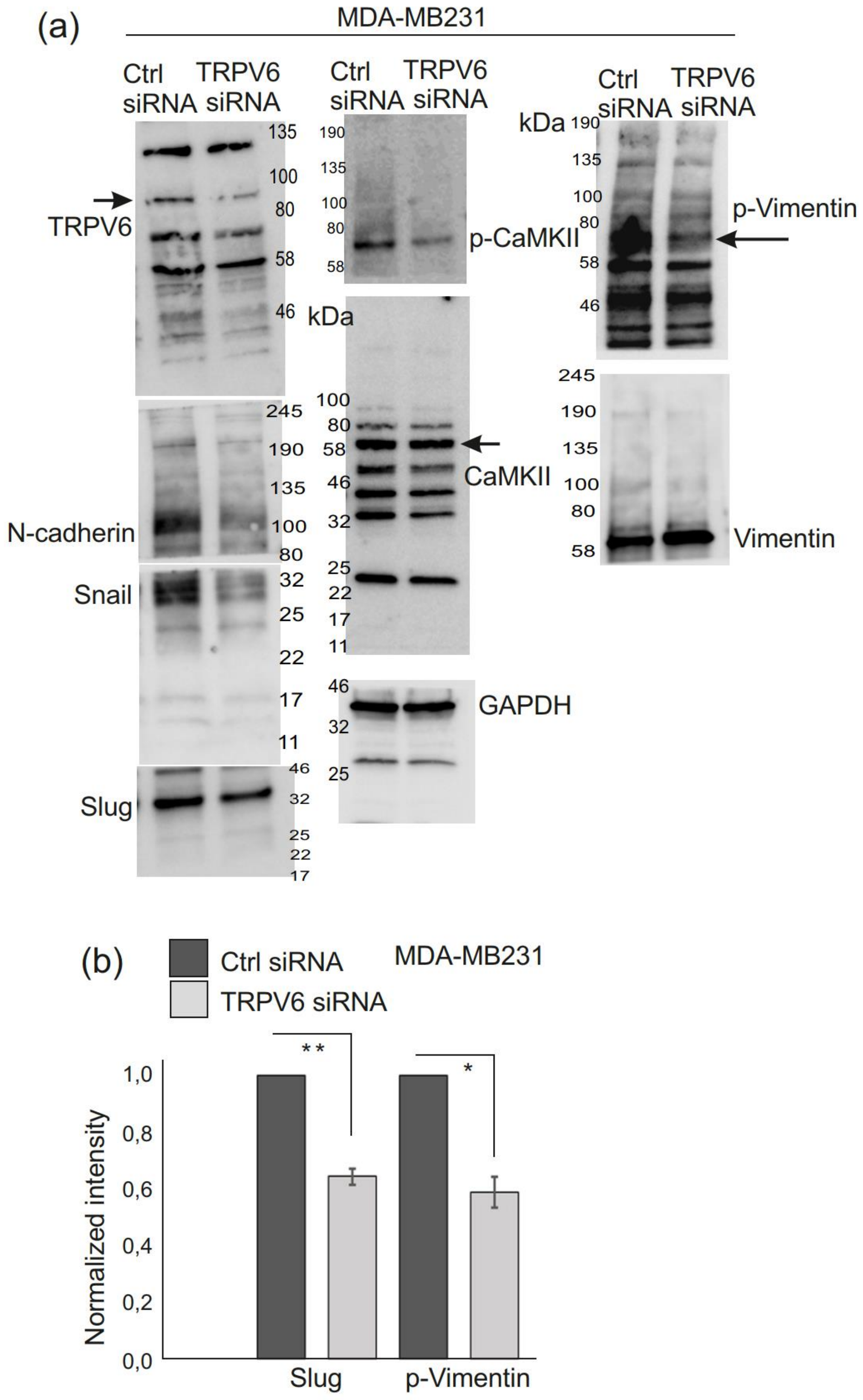

Figure S8. TRPV6 is linked to the expression of EMT-associated proteins in MDA-MB-231 breast cancer cells. a) Depletion of TRPV6 from MDA-MB231 breast carcinoma cells by specific

siRNAs. Levels of specific EMT-associated proteins were evaluated from the cellular lysates in Western blotting experiments. GAPDH was used as a loading control. Uncropped Western blots are presented and weight markers (kDa) for the indicated proteins are shown next to the blots. **b)** Quantification of vimentin and slug levels, related to Fig. S8a. Intensity values were measure in ImageJ and intensity values for vimentin and slug were divided with the corresponding GAPDH values. Control sample values were normalized to 1. Mean (+/- SEM) is shown. n= 3. *P<0.05; **P<0.01 (Paired t-test).

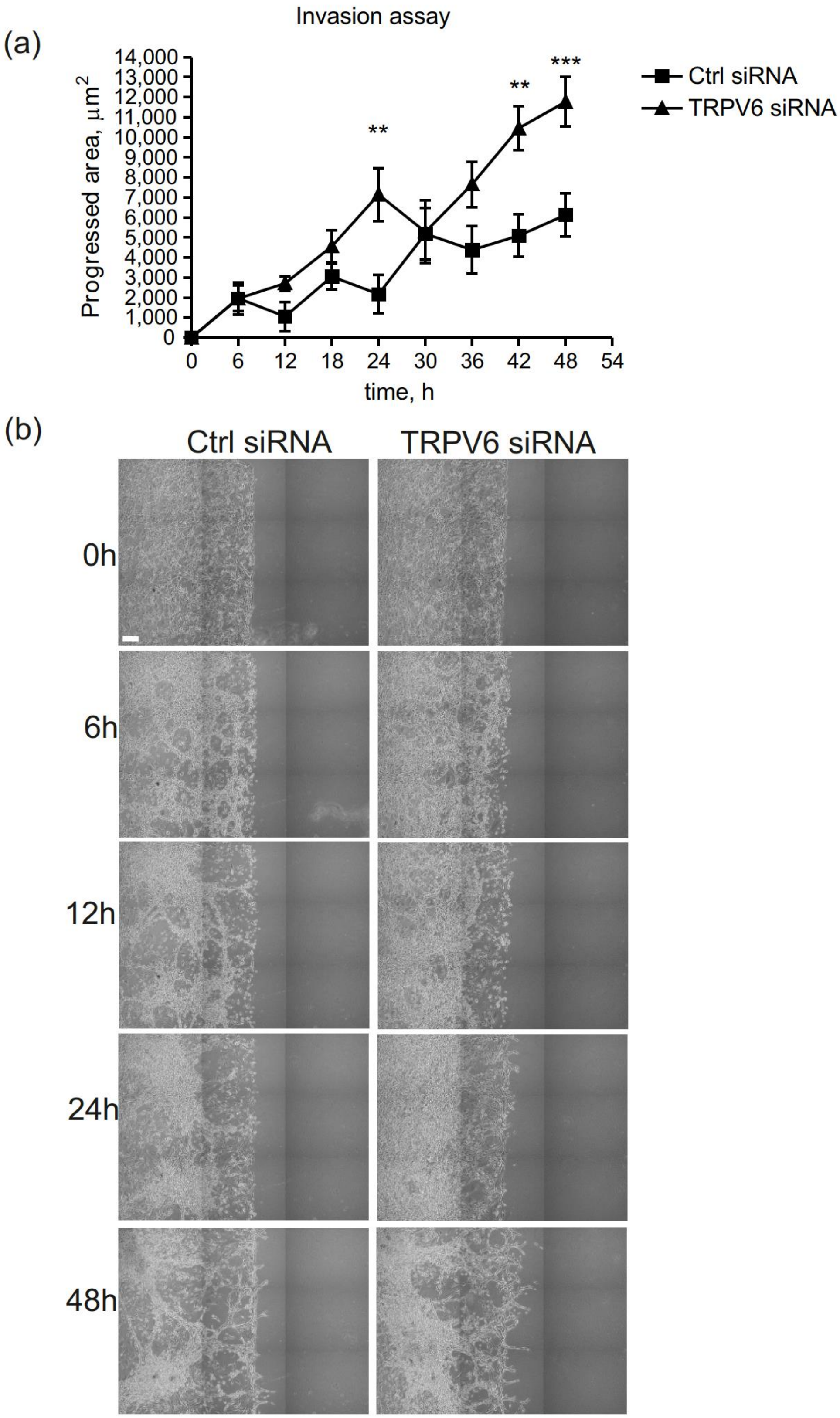

Figure S9. TRPV6 expression and invasion of MDA-MB-231 breast cancer cells. a) Invasion assay in Matrigel. Control- and TRPV6 siRNA-treated MDA-MB-231 cells were monitored in the assay. Results are presented as mean (+/- SEM). n= 4. **P<0.01, ***P<0.001, Two-way ANOVA,

Bonferroni's post-test. **b)** Representative brightfield images of the ctrl – and TRPV6 siRNA cells at given time points, along the invasion assay. Bar 250 um.
